# Molecular signature of the putative stem/progenitor cells committed to the development of the bovine mammary gland at puberty

**DOI:** 10.1038/s41598-018-34691-2

**Published:** 2018-11-01

**Authors:** Laurence Finot, Eric Chanat, Frederic Dessauge

**Affiliations:** UMR 1348 PEGASE, Agrocampus Ouest, INRA, Saint-Gilles, France

## Abstract

Milk production is highly dependent on the extensive development of the mammary epithelium, which occurs during puberty. It is therefore essential to distinguish the epithelial cells committed to development from the related epithelial hierarchy. Using cell phenotyping and sorting, we highlighted four cell sub-populations within the bovine mammary gland at puberty. The CD49_f_^high^CD24^neg^ cells expressing CD10, *KRT14*, *vimentin* and *PROCR* corresponded to cells committed to the basal lineage. The CD49_f_^low^ sub-population contained two cell subsets (CD49_f_^low^CD24^neg^ and CD49_f_^low^CD24^pos^). Both subsets expressed hormone receptors including *ER*, *PR* and *PRLR*, as well as ALDH1 activity but only the CD49_f_^low^CD24^pos^ subset expressed *ELF5*. These data indicated that the CD49_f_^low^ sub-population is mainly composed of cells displaying a luminal phenotype and that this population comprises two luminal cell subsets, namely the CD24^neg^ and CD24^pos^ cells, likely committed to ductal and alveolar lineage, respectively. The putative mammary stem cell (MaSC) fraction was recovered in the CD49_f_^high^CD24^pos^ sub-population which were shown to form mammospheres *in vitro*. These cells differentially expressed CD10, *KRT14* and *KRT7*, suggesting the existence of several putative MaSC sub-fractions. In-depth characterization of these epithelial sub-populations provides new insights into the bovine mammary epithelial cell lineage and suggests a common developmental lineage in mammals.

## Introduction

The mammary gland undergoes dynamic morphological changes over the lifetime of female mammals. At birth, bovine mammary parenchyma consists of a rudimentary duct network connected to a small cisternal cavity. At the onset of puberty, the mammary rudiment develops and starts to expand into the stroma upon stimulation by the ovarian steroid hormones, including estradiol and progesterone, and by growth factors^1^. Ductal elongation occurs through the growth, development, and subsequent extension of terminal ductal lobular units (TDLU) in a process referred to as branching morphogenesis. Bovine mammary TDLUs initially consist of solid cords of epithelial cells that penetrate into the stroma. As these cords extend into the mammary fat pad, lateral outgrowths emerge. This parenchymal development continues through puberty, until the mammary fat pad becomes filled. In addition, during gestation, the tissue continues its differentiation with the formation of lobulo-alveolar structures and the maturation of TDLUs in response to circulating hormones, notably prolactin. At the end of its development, the mammary epithelium has the appearance of an elaborate tree of ducts and alveoli. After parturition, the alveolar epithelium starts to be fully functional, with mammary epithelial cells secreting milk proteins into the lumen of the alveoli for lactation^2^.

The ability of the mammary gland to undergo many cycles of lactation, with their stages of tissue proliferation and involution, suggests that the epithelial compartment contains resident cells capable of generating the entire epithelial architecture. Evidence for the existence of mammary stem cells (MaSCs) has been primarily derived from transplantation studies with murine mammary tissues. These studies revealed that the ductal architecture could be regenerated *in vivo* when isolated parenchymal explants were transplanted into cleared mammary fat pads^3–5^. More recent assays showed that an entire and functional mammary gland can be reconstituted from the transplantation of the progeny of a single “stem-like” cell^6,7^. Since these pioneering demonstrations, many studies in murine and human species have focused on identifying and isolating MaSC populations in order to establish the hierarchical cell organization and the molecular players in the regulation of the epithelium^8,9^. The epithelial hierarchy can be described as a pyramidal setup of the epithelial cell populations with stem cells at the apex and differentiated mature cells at the base of the pyramid. Between these two cell populations are the multiple progenitors that originate from the division and activation of stem cells and that progressively differentiate into mature cell lineages. Of note, the mammary structures are described as being composed of two major lineages: the luminal and basal cells, the latter including the myoepithelial cells. Luminal and basal cells can be distinguished by either their location in the epithelial structure or their protein expression profiles. Cells of these two lineages are considered immature during development as compared to the differentiated (mature) cells that constitute the functional secretory tissue.

In contrast, in bovines, only a few groups have attempted to elucidate the epithelial hierarchy *via* the identification of progenitor/stem cell populations^10,11^. We recently participated in this research effort by providing original data on the mammary epithelial hierarchy committed to lactation during a lactation cycle in bovines^12^. In this study, we used flow cytometry analysis and fluorescence activated cell sorting based on the expression of classic markers previously identified in the murine, human and bovine species. These markers are cell surface proteins, including the cluster of differentiation (CD) 24 (heat-stable antigen), CD29 (ß1-integrin) or CD49f (α6-integrin), and CD10^13,14^. These approaches led us to isolate putative populations of MaSCs, a prerequisite for further study of these target cell populations.

Research on MaSC biology in dairy mammals is important and relates to their potential use to improve animal robustness through the enhancement of lactation efficiency and infection resistance. A better understanding of the epithelial hierarchy at each developmental stage is therefore a prerequisite for the optimization of lactation in cows. Until now, literature describing the epithelial cell populations at key developmental stages (after puberty) and the regulators governing the bovine epithelial hierarchy has been scant. In this context, our study aims to further characterize the cells that make up the epithelial lineage at the branching morphogenesis stage in order to provide new insights into the epithelial hierarchy.

## Results

### Discrimination between cell sub-populations within the mammary epithelium of pubertal cows using the cell surface markers CD49_f_, CD24 and CD10

Since puberty is a key period of mammary gland development during which the different epithelial lineages, basal/myoepithelial and luminal cells, are committed to the process of branching morphogenesis and are identifiable, we used mammary gland samples from pubertal cows for our study.

**Figure 1:**
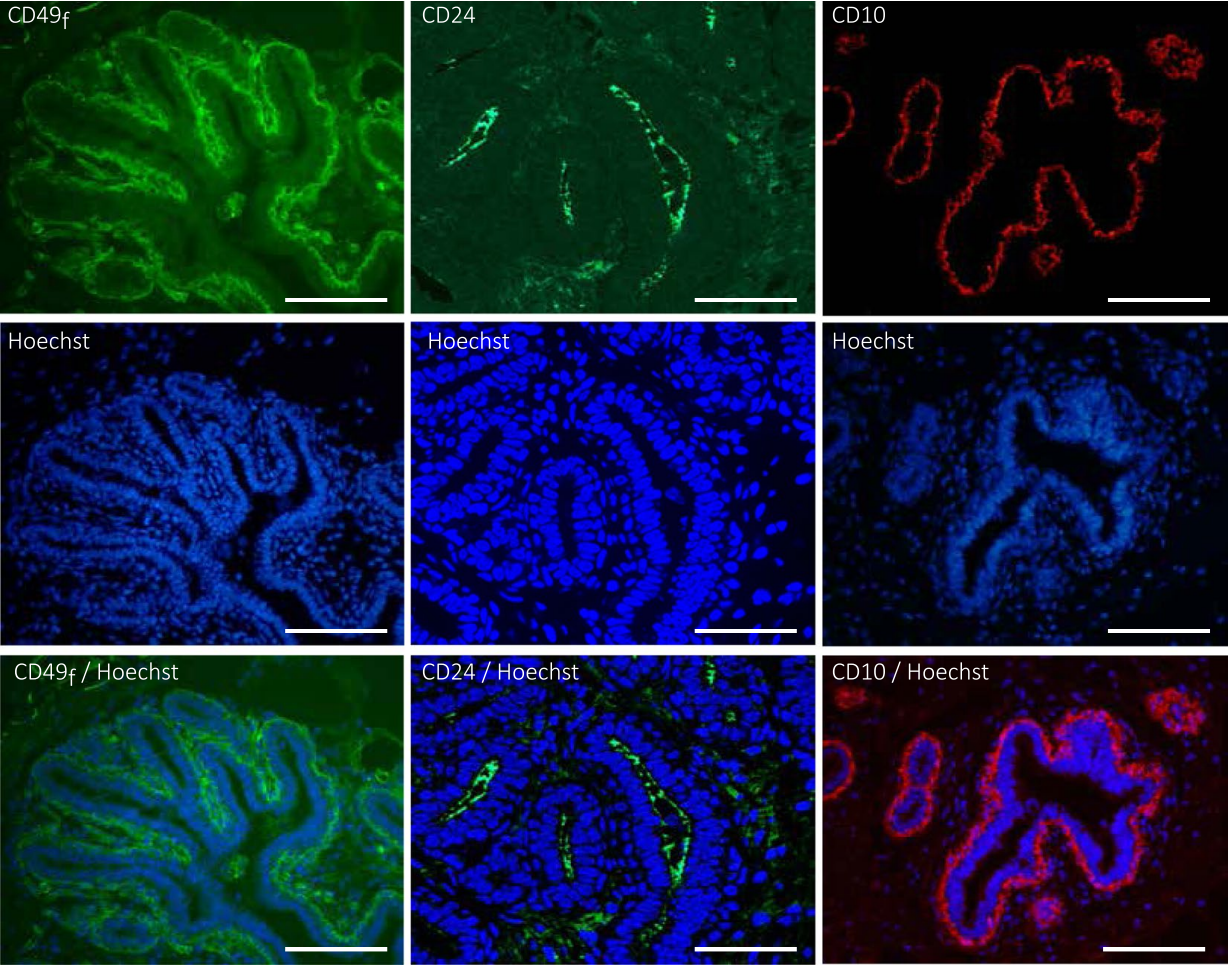
The cell surface markers CD49_f_, CD24 and CD10 are located in the luminal and basal cells within the ductal mammary epithelium of cows at puberty. Cryo- (CD49_f_ and CD24) and paraffin sections (CD10) from the mammary tissue of pubertal cows were processed for immunofluorescence for the indicated antigens. Nuclei were counterstained with Hoechst 33342. Note that the CD24 images were obtained with an Apotome microscope. The basal membrane of the outer cell layer of the epithelium was highly stained for CD49_f_ whereas luminal cells were weakly stained (left panels, green). CD24-positive cells were located within mammary epithelial ductal structures (middle panels, green). Antibodies against CD10 nicely stained the outer cells of the developing ductal structures (right panels, red). Images are representative of three cows. Scale bars, 100 µm.

In agreement with this, tissue staining with hematoxylin and eosin showed numerous neo-formed ductal and alveolar structures constituting an epithelium that largely formed the mammary parenchyma (Fig. S1). To identify the cell sub-populations of the epithelial lineages acting in the building of this parenchyma in the most exhaustive way possible, we focused our analysis on three cell surface markers that are well known to be specific for mammary epithelial cells: CD49_f_, CD24 and CD10. To validate our approach, we first analyzed the *in situ* localization of the cells expressing these markers by immunofluorescence. As shown in Fig. 1, cells of the ductal trees at the origin of future TDLUs were clearly stained by anti-CD49_f_ antibodies (Fig. 1, left panels). The outer cells of these epithelial structures formed a monolayer and were strongly stained at their basal side, whereas the inner cells were weakly stained. In contrast, CD24 was expressed apically by epithelial cells located in the lumen of ductal structures in development (Fig. 1, middle panels). As to CD10, which has been described as a cell surface marker of basal cells, it was clearly expressed by cells surrounding the developing duct structures (Fig. 1, right panels). In this case, stained cells were exclusively localized to the outer epithelium layer, or sometimes appeared in small clusters (see the little structure at the top right of the image; Fig. 1, right panels). These immuno-histological results having confirmed the relevance of using these markers, we decided to evaluate the proportion of each cell sub-population of the mammary tissue expressing them by flow cytometry.

As shown in the cytometric profile of CD49_f_ expression (Fig. S2a, upper plot), 62% (±1.8%) of total single cells prepared from the mammary tissue of pubertal cows were CD49_f_^pos^ cells. Moreover, it was possible to distinguish two distinct sub-populations within these cells: the CD49_f_^low^ (38%) and CD49_f_^high^ (24%) sub-populations. To further identify the cell types that compose the mammary gland tissue of the pubertal cow, total single cells were sorted based on CD49_f_ expression. A set of proteins known to be specifically expressed in the epithelial lineage was then quantified in both negative and positive cell sub-populations by Western blotting (Fig. S3). What was highly noticeable was the higher expression level of all epithelial lineage protein markers in the CD49_f_^pos^ cells as compared to the CD49_f_^neg^ cells (Fig. S3a,b). Only the CD49_f_^pos^ cells expressed the epithelial cadherin (CDH1, Fig. S3a, left graph), the basal marker keratin (KRT) 14 and the myoepithelial marker alpha-smooth muscle actin (αSMA), as well as the luminal KRT7, KRT19 and KRT18 (see also Fig. S4 for the *in situ* lineage-specific localization of KRT). Finally, these cells significantly overexpressed the basal marker CD10 (Fig. S3b). Altogether, these data strongly suggested that CD49_f_ cell sorting allowed the recovery of epithelial cells of both basal and luminal origins.

When cells were analyzed for CD24 expression, a unique heterogeneous population of CD24^pos^ cells was observed (Fig. S2a, middle plot). It accounted for 32% (±9.8%) of total single mammary cells. Western blotting showed that the epithelial marker CDH1 was expressed in both CD24^neg^ and CD24^pos^ cells (Fig. S3b) but was much more abundant in the latter cells. As a whole, the CD24^neg^ cells preferentially expressed the basal markers, i.e., CD10, αSMA and KRT14, whereas the luminal markers were more highly expressed in the CD24^pos^ cells. Indeed, both CD24^neg^ and CD24^pos^ cells expressed KRT7, KRT18 and KRT19, but all the luminal keratins were expressed at significantly higher levels in the CD24^pos^ population (Fig. S3a, middle graph and Fig. S3b). We concluded that CD24 is a marker that allows the distinction of epithelial sub-populations within the basal and the luminal lineage.

Finally, we identified two cell sub-populations expressing CD10 (CD10^low^ and CD10^high^), the sum of which accounted for 41% (±7.7%) of total mammary cells (Fig. S2a, bottom plot). KRT14 was only present in the CD10^pos^ cells (Fig. S3a, right graph and Fig. S3b). In addition, αSMA was almost 6-fold more abundant in the CD10^pos^ population than in the CD10^neg^ population (15.2% ± 4% *vs* 3.1% ± 0.5%). Interestingly, the luminal KRT19, KRT18 and KRT7 were expressed in both the CD10^neg^ and CD10^pos^ cell sub-populations with no significant difference, except for KRT7 which was expressed at 6-fold higher level in the CD10^pos^ cells (3.79% ± 0.97% *vs*. 0,55% ± 0.18%). In summary, our data confirm that CD10 expression is characteristic of basal cells, making it a pertinent marker to discriminate the basal lineage from the luminal lineage.

### Determination of the cell sub-populations involved in mammary gland development at puberty

**Figure 2:**
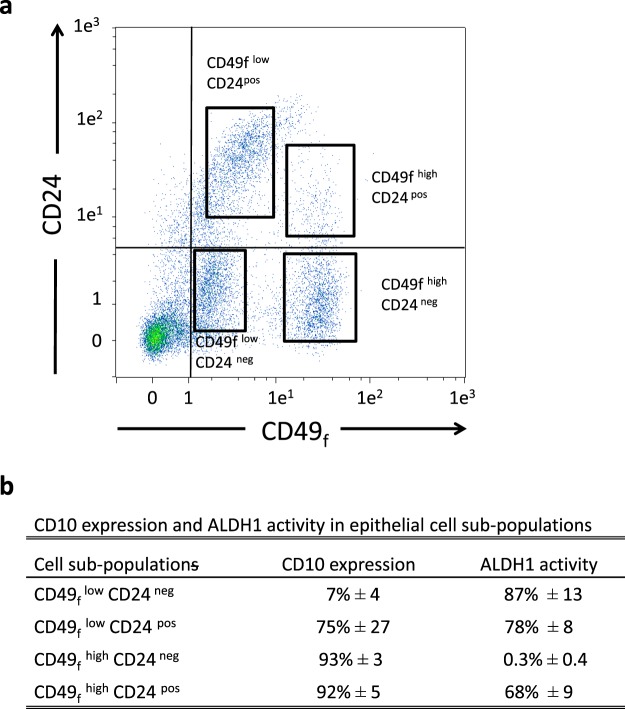
Sub-populations of epithelial cells exhibit distinct lineage types in the developing bovine mammary gland. (**a**) Cells dissociated from pubertal bovine mammary tissue were co-stained with anti-CD49_f_ -FITC (CD49_f_) and anti-CD24-APC (CD24) antibodies and analyzed by flow cytometry. The gating for each control immunoglobulin isotype is indicated by the solid lines. (**b**) Cells expressing low or high levels of CD49_f_ and/or CD24 were subjected to flow cytometry analysis for either CD10 expression or ALDH1 activity. The mean percentages of cells (±SEM) expressing CD10 or showing ALDH1 activity in each sub-population from three independent experiments (3 cows) are summarized in a table. Abbreviations: ALDH1, Aldehyde dehydrogenase 1.

To further delineate the different cell sub-populations involved in the development of the mammary gland in pubertal cows, we analyzed all combinations of cell co-staining with CD49_f_, CD24 and CD10 by flow cytometry. Co-staining for CD49_f_ and CD24 revealed four distinct positive cell sub-populations in addition to the double-negative population (Fig. 2a). The majority of the cells was CD49_f_^pos^CD24^neg^ (42% ± 0.8% of total cells) and equally distributed into CD49_f_^low^ and CD49_f_^high^ cells (see Table 1 for the relative proportion of each sub-populations). The CD49_f_^pos^CD24^pos^ sub-populations represented 20% (±3.7%) of total single cells with a large proportion of CD49_f_^low^CD24^pos^ cells (Fig. 2a and Table 1). Interestingly, each of these sub-populations (CD49_f_^low^CD24^pos^, CD49_f_^low^CD24^neg^ and CD49_f_^high^CD24^neg^) approximately accounted for one third of the total CD49_f_^pos^ cells (see Fig. S5). Finally, we found that only 2% (±0.1) of total single cells were CD49_f_^neg^CD24^pos^. Co-staining for CD49_f_ and CD10 revealed five distinct sub-populations (Fig. S2b, middle plot, Table 1). Finally, co-staining for CD10 and CD24 revealed heterogeneous sub-populations (Fig. S2B, bottom plot, Table 1). Altogether, these data highlighted the multiple cell sub-populations present within the mammary tissue during pubertal development.

**Table 1:** Results of flow cytometry analysis for CD49f, CD24 and CD10 expression in mammary gland of cows.

Monostained Populations	% ± SEM
**CD49**_**f**_ **Populations**	
CD49_f_^neg^	37.4 ± *1*.*8*
CD49_f_^pos^	62.6 ± *1*.*8*
**D24 Populations**	
CD24^neg^	67.4 ± *9*.*2*
CD24^pos^	32.5 ± *9*.*8*
**CD10 Populations**	
CD10^neg^	62.9 ± *13*.*7*
CD10^pos^	41.3 ± 7.7
**Doublestained Populations and Subpopulations**	
**CD49**_**f**_ **/CD24 Populations**	
CD49_f_^neg^ CD24^neg^	35.3 ± *1*.*7*
CD49_f_^neg^ CD24^pos^	2.2 ± 0.1
CD49_f_^pos^ CD24^neg^	41.9 ± *2*.*7*
CD49_f_^low^ CD24^neg^	20.8 ± *0*.*8*
CD49_f_^high^ CD24^neg^	20.8 ± *2*.*3*
CD49_f_^pos^ CD24^pos^	20.6 ± *3*.*7*
CD49_f_^low^ CD24^pos^	16.8 ± *3*.*2*
CD49_f_^high^ CD24^pos^	3.4 ± *0*.*4*
**CD49**_**f**_ **/CD10 Populations**	
CD49_f_^neg^ CD10^neg^	23.4 ± 3.*8*
CD49_f_^neg^ CD10^pos^	14.2 ± *4*.*4*
CD49_f_^pos^ CD10^neg^	25.8 ± *3*.*7*
CD49_f_^low^ CD10^neg^	20.7 ± *3*.*2*
CD49_f_^high^ CD10^neg^	2.1 ± *0*.*3*
CD49_f_^pos^ CD10^pos^	36.5 ± *2*.*5*
CD49_f_^low^ CD10^pos^	13.7 ± *1*.*4*
CD49_f_^high^ CD10^pos^	17.1 ± *3*.*9*
**CD10/CD24 populations**	
CD10^neg^ CD24^neg^	40.9 ± 1.*9*
CD10^neg^ CD24^pos^	15.4 ± *1*.*8*
CD10^pos^ CD24^neg^	23 ± *6*.*2*
CD10^pos^ CD24^pos^	20 ± *6*.*4*

Data of cellular populations and sub-populations are expressed as the mean percentage of cells ± SEM from three independent experiments (3 cows).

### Characterization of the cell sub-populations composing the mammary epithelial hierarchy

**Figure 3:**
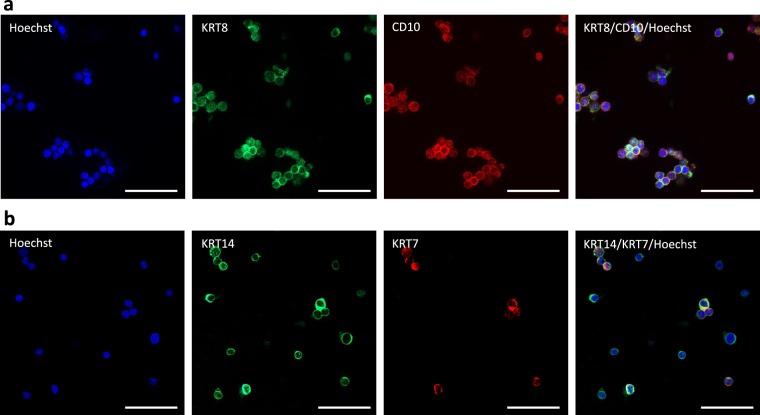
Analysis of luminal and basal marker protein expression highlights heterogeneity of the MaSC fraction. Cells dissociated from pubertal bovine mammary tissue were sorted according to the level of expression of both CD49_f_ and CD24 and analyzed by immunofluorescence. (**a**) CD49_f_^low^ CD24^pos^ cells were co-stained with anti-CD10 and -KRT8 antibodies. (**b**) CD49_f_^high^ CD24^pos^ cells were co-stained with anti-keratin (KRT) 14 and KRT7 antibodies. Nuclei were counterstained with Hoechst 33342. Scale bars, 100 µm.

Since mammary stem cells and progenitors were reported to belong to a subset of CD49_f_^pos^CD24^pos^ cells, we decided to further investigate the phenotype of this cell sub-population. We found that the CD49_f_^low^CD24^neg^ cells were predominantly negative for CD10 whereas almost all CD49_f_^high^ cells expressed CD10 at high level (Fig. 2b). Within the CD49_f_^low^CD24^pos^ sub-population, 75% of the cells were positive for CD10. To confirm the double lineage of this sub-population, we analyzed sorted cells by immunofluorescence and found that the CD49_f_^low^CD24^pos^ cells co-expressed CD10 and KRT8, a luminal marker protein (Fig. 3a). Similarly, we evaluated the activity of aldehyde dehydrogenase 1 (ALDH1) in the aforementioned CD49_f_^pos^ sub-populations. Indeed, ALDH1 activity has been previously identified as a marker of luminal cells and it has been shown to distinguish progenitor from mature mammary luminal cells in different species^15^. We found that 78 to 87% of the CD49_f_^low^ cells, namely the CD49_f_^low^CD24^neg^ and the CD49_f_^low^CD24^pos^ cells, exhibited ALDH1 activity, as well as 68% of the CD49_f_^high^ CD24^pos^ cells (Fig. 2b). It is therefore reasonable to assume that these three sub-populations belong or are related to the luminal lineage.

**Figure 4:**
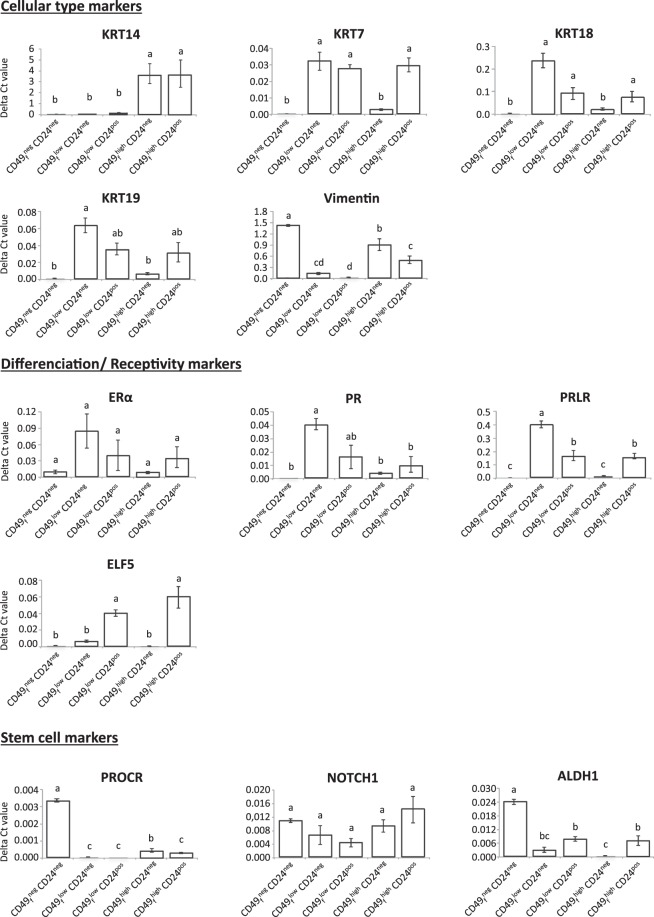
Gene expression levels in epithelial cell sub-populations. Cells dissociated from pubertal bovine mammary tissue were co-stained with anti-CD49_f_ -FITC (CD49_f_) and anti-CD24-APC (CD24) antibodies and five sub-populations were sorted. The level of expression of the indicated genes was measured in each sub-population by RT-qPCR. Data are expressed as the mean of Delta Ct calculation ± SEM from three independent experiments (3 cows). Different letters (a–d) indicate significant differences. Abbreviations: ALDH1, Aldehyde dehydrogenase 1; ELF5, E74-like factor 5; ERα, Estradiol receptor alpha; KRT, keratin; NOTCH1, Notch homolog 1, translocation-associated; PR, Progesterone receptor; PRLR, prolactin receptor; PROCR, Protein C receptor.

We next investigated the expression of target genes by RT-qPCR (Fig. 4). Hormone receptivity of the pubertal mammary tissue was also assessed beforehand by immunofluorescence (Fig. S6a). These analysis revealed the expression of the progesterone (PR) and estradiol (ERα) receptors by the epithelial cells (22% ± 2.4% and 11% ± 1% of total cells for the PR and ERα-stained cells, respectively) (Fig. S6b). We found that the genes known to be expressed by stromal cells, namely *vimentin*, *ALDH1* and the *Protein C receptor* (*PROCR*) were significantly more expressed in the CD49_f_^neg^CD24^neg^ sub-population. In contrast, this sub-population under-expressed genes of the KRT family and the differentiation/receptivity markers. On the other hand, the two CD49_f_^low^ sub-populations expressed higher levels of *KRT19*, *KRT18 and KRT7* as compared to the CD49_f_^neg^ sub-population, confirming their luminal origin. However, the CD49_f_^low^CD24^neg^ and CD49_f_^low^CD24^pos^ sub-populations presented differences in KRT expression (2 and 2.6-fold more abundant for *KRT19* and *KRT18*, respectively, in the CD49_f_^low^CD24^neg^ sub-population than in the CD49_f_^low^CD24^pos^ sub-population) and in their hormonal receptivity (2.5-fold more abundant for *PR* and *prolactin receptor* (*PRLR)* in the CD49_f_^low^CD24^neg^ sub-population than in the CD49_f_^low^CD24^pos^ sub-population). The CD49_f_^low^CD24^neg^ sub-population was characterized by expression of the three luminal keratins and of both *PR* and *PRLR*. The CD49_f_^low^CD24^pos^ sub-population especially expressed the luminal *KRT7*, the stemness markers *ALDH1* and the receptivity markers *PR* and *E74-like factor 5 (ELF5)*. As for the CD49_f_^high^ sub-populations, they significantly expressed *KRT14*, confirming their basal origin. Finally, the CD49_f_^high^CD24^neg^ sub-population was characterized by a moderate abundance of the *vimentin* and *PROCR* genes whereas the CD49_f_^high^CD24^pos^ sub-population expressed the *KRT7*, *ALDH1 and ELF5* genes. Cells of this latest sub-population were also found to express the basal markers CD10 and KRT14, inferring some concerns about its homogeneity. In line with this, immunofluorescence analysis of sorted cells confirmed that the CD49_f_^high^CD24^pos^ sub-population contained both cells expressing only KRT14, and cells co-expressing KRT14 and KRT7 (Fig. 3b). In conclusion, each CD49_f_ CD24 sub-population exhibited a unique phenotype and molecular signature which allowed them to be catalogued in a lineage type.

**Figure 5:**
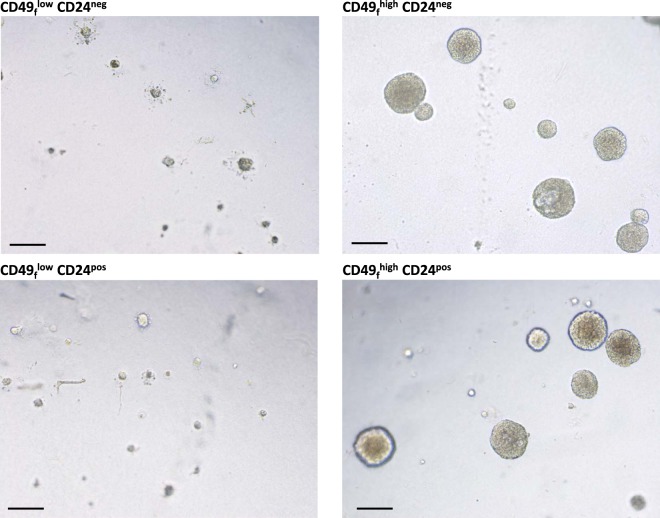
CD49_f_^high^ sub-populations displayed functional properties to form mammospheres. Cells dissociated from pubertal bovine mammary tissue were sorted according to the level of expression of both CD49_f_ and CD24. The resulting epithelial cell sub-populations were cultured in Matrigel during 7 days to assess the formation of mammospheres. Images are representative of three independent experiments (3 cows). Scale bars, 200 µm.

It was shown that stem cells survive, proliferate and form spheres of cells (called mammospheres) when grown in three-D culture in the presence of Matrigel. In fact, the formation of mammospheres now accounts for a functional assay of stem cells. We therefore tested the ability of the sorted epithelial cell sub-populations to form spheres (Fig. 5). After 7 days of culture in Matrigel, both the CD49_f_^low^CD24^neg^ and CD49_f_^low^CD24^pos^ sub-populations failed to form mammospheres. In striking contrast, the CD49_f_^high^CD24^pos^ and CD49_f_^high^CD24^neg^ cells formed well-shaped mammospheres of 186 µm (±12 µm) and 173 µm (±8 µm) diameters, respectively.

## Discussion

After puberty, each estrous cycle is accompanied by periods of enhanced cell proliferation and differentiation in the mammary gland until the fat pad is filled with parenchymal tissue. Therefore, this post-pubertal stage is a key period to search for and identify the most epithelial cell categories, including progenitor cells. Based on the literature, the analysis of the expression of the specific cell surface markers CD49_f,_ CD24 and CD10 in bovine mammary epithelial cells at puberty by flow cytometry allowed for the identification and isolation of prospective key cell sub-populations. Of course, it was of the utmost interest to further analyze the molecular signatures of these sub-populations to improve our knowledge of the mammary epithelial cell lineage.

We first found that the CD49_f_^high^CD24^neg^ sub-population expressed *KRT14*, a well-known marker of the basal lineage classically associated with basal/myoepithelial cells^16,17^. This sub-population also substantially expressed CD10, another marker of basal cells^17^. Finally, immunohistological observations revealed that the cells of the outer epithelium layer were strongly stained at their basal side by anti-CD49_f_ antibodies. In summary, these data indicate that the CD49_f_^high^CD24^neg^ sub-population is from the basal lineage. This is in agreement with a previous bovine study on the characterization of the epithelial cells present in the mammary gland a few months after birth^10^. More recently, this group reported that, at early developmental stages, the basal cells were CD49_f_^pos^CD24^neg^, and specified that their phenotype was CD49_f_^high^CD24^neg^^18^. In the present study, we further characterized this basal CD49_f_^high^CD24^neg^ sub-population by, notably, studying the expression of the *vimentin* and *PROCR* genes. Indeed, vimentin filaments are expressed, *inter alia*, in the basal epithelial cell population of the mammary gland^19^ and it has recently been demonstrated that *vimentin* deficiency in *vimentin* KO mice affects mammary ductal development by altering progenitor cell activity^19^. This suggested a regulatory role of vimentin in the basal MaSC/progenitor cell population. The fact that the bovine CD49_f_^high^CD24^neg^ cell analysed here expressed *vimentin* therefore suggest that they might be progenitor cells. This is also supported by the observation that these cells also expressed high levels of *PROCR*. Indeed, this gene which was found to be relatively abundant in the basal cells of murine mammary epithelium was suggested to be a marker of mammary stem cells^20^. This possibility was previously envisioned in a model of human breast cancers in which the receptor was one of the molecular markers for stem/progenitor-like populations^21^. However, our current knowledge of the bovine model does not allow us to firmly conclude whether the CD49_f_^high^CD24^neg^ cells account for the basal progenitor cells, but only that they are most likely committed to basal/myoepithelial lineage.

We observed by immunofluorescence that the cells localized into the inner epithelium layer expressed low levels of CD49_f_. Also, our cytometric profiles showed that two mammary epithelial cell sub-populations expressed low levels of CD49_f_, namely the CD49_f_^low^CD24^neg^ and CD49_f_^low^CD24^pos^ cell sub-populations. This is in agreement with the aforementioned study in bovines and with studies in mouse, in which the luminal population was reported as being CD49_f_^low^ ^18,22,23^. In addition to these data, we showed by Western blotting that KRT19, KRT18 and KRT7 were expressed by the CD49_f_^pos^ cells, including the CD49_f_^low^ cells. The abundance of these luminal KRTs was also demonstrated at the mRNA level in the two CD49_f_^low^ sub-populations. These data confirm that the CD49_f_^low^ sub-populations belong to the luminal lineage.

Many studies have reported that mammary development at puberty is triggered by the main steroid hormones estradiol and progesterone (for review see^2^). Indeed, experiments with *PR*-deficient mice demonstrated that, at puberty, progesterone is not essential for ductal elongation but is critical in inducing side-branching^24^. This observation suggests that progesterone, independent from estradiol, could intervene late in branching morphogenesis to promote side branching and later in the formation of lobulo-alveolar structures^25^. Moreover, it has been found that a large number of luminal cells are PR-positive in adult virgin mice at an advanced stage of puberty^26^. We also observed that PR staining is restricted to luminal cells. As to the key role of prolactin, it has been found that deletion of the *PRLR* in mice resulted in defects in side branching and further alveolar formation^27^. Conversely, overexpression of prolactin in mice has been shown to increase lateral ductal budding and to increase epithelial progenitor sub-populations^23^. Here, we found that both the CD49_f_^low^CD24^neg^ and CD49_f_^low^CD24^pos^ sub-populations expressed the hormone receptors (*ER*, *PR* and *PRLR*). Additionally, we observed that the CD49_f_^low^CD24^pos^ sub-population expressed *ELF5* in contrast to the CD49_f_^low^CD24^neg^ sub-population. Interestingly, the transcription factor ELF5 is known to orient the fate of luminal cells during alveogenesis^28^. In the murine model, the CD49_f_^low^CD24^neg^ cell sub-population was described to represent the ductal cell population^29^. Altogether, our data indicated that the CD49_f_^low^ population is mainly composed of cells displaying a luminal phenotype and that this population comprises two major luminal subsets, namely CD24^neg^ and CD24^pos^, likely committed to ductal and alveolar lineage, respectively.

Furthermore, we found that both CD49_f_^low^CD24^neg^ and CD49_f_^low^CD24^pos^ sub-populations exhibited ALDH1 activity, a feature that characterizes the differentiation status of the luminal cells. Indeed, a previous study in human mammary gland demonstrated that ALDH1 activity was upregulated at the transition of progenitor cells into the luminal lineage^15^. ALDH1 activity has also been used in the bovine model to define luminal-restricted progenitors^30^ and in the mouse model to distinguish the relatively undifferentiated luminal progenitors from the differentiated ones^31,32^. From these reports and our data, we could hypothesise that the two CD49_f_^low^ sub-populations are luminal progenitors. However, further investigations are required to confirm the progenitor commitment of these sub-populations.

In many species, whether human, murine or bovine, the MaSC population has been described as being CD49_f_^high^CD24^pos^^9,33,34^. Here, this cell sub-population represented 3.4% of total mammary cells. This relatively small percentage was consistent with what is usually reported for the MaSC-enriched fraction in the literature (5% of total mammary cells in mice and 2.43% in post-pubertal bovines^35^). Recently, we showed that the proportion of CD49_f_^high^CD24^pos^ cells in the bovine lactating mammary gland range from 0.7% to 3.3%^12^. In the present study, we found that this sub-population also expressed the two basal markers CD10 and *KRT14*. This was consistent with the observation that MaSCs appeared localized to the basal compartment, sharing characteristics with the surrounding basal cells^36–38^. As observed previously^8^ and confirmed here, the CD49_f_^high^CD24^pos^ sub-population formed mammospheres when cultured in the presence of Matrigel. The above considerations suggest that the CD49_f_^high^CD24^pos^ sub-population is the putative MaSC fraction. Since this fraction exhibited heterogeneous phenotypes with variable ALDH1 activity, KRT7 and KRT14 expression, one can conclude that the CD49_f_^high^CD24^pos^ sub-population contains several MaSC sub-fractions. This notion has recently been raised in an elegant study of the murine MaSCs^39^ in which the dynamics of branching morphogenesis were monitored by highlighting the behaviour of the different lineage-committed MaSCs using a “confetti” cell strategy. Indeed, it was concluded that a pool of MaSCs is engaged in the development of the tissue whereas another stays quiescent. By analogy, we can hypothesize that the putative MaSC sub-populations exhibiting ALDH1 activity and KRT14/KRT7 expression represent the lineage-restricted “activated” MaSC engaged in epithelial development.

Even if it could be confusing to compare mammary epithelial cell lineages between species, notably because investigators regularly use different cell markers^40^, the mammary epithelial cell lineages we described here shares many common characteristics with that proposed for the murine model^9^. In agreement with this, our data lead us to hypothesize that the putative bovine MaSC fraction is most likely the CD49_f_^high^CD24^pos^ sub-population, as proposed to date in several reports^34,40,41^. Refining our knowledge on MaSC in dairy animals is of utmost importance in order to properly manipulate MaSC differentiation potential and/or expansion. Indeed, it would provide means to increase the robustness traits of dairy cows by increasing the number of fully functional mammary secretory cells and by improving the capacity of replacement of senescent or damaged cells during lactation.

## Materials and Methods

All the animal procedures were discussed and approved by the CNREEA No. 07 (Local Ethics Committee in Animal Experiment of Rennes) in compliance with French regulations (Decree No. 2013–118, February 07, 2013).

### Animals

The Holstein cows (*bos taurus*) used in this study were housed at the experimental farm of Méjusseaume INRA-Rennes (France). The pubertal cows were sacrificed at 17 months of age at the slaughterhouse of Gallais Viande (Montauban-de-Bretagne, France) following standard commercial practices. The mammary glands were collected at the time of slaughter and immediately transported on ice to the laboratory to be sampled for further analyses.

### Mammary tissue sampling

Total parenchyma of the mammary gland was dissected and sampled. Samples destined for tissue dissociation were manually cut into small explants (≈1 mm^3^), suspended in 90% fetal bovine serum (10270-106; Gibco Invitrogen Saint Aubin, France)/ 10% dimethyl sulfoxide (DMSO, D2650, Sigma-Aldrich, Saint-Quentin Fallavier, France), and stored at −150 °C. For immunohistological analysis, tissue pieces (≈5 mm^3^) were fixed in 4% paraformaldehyde (FOR007OAF59001, VWR, Fontenay-sous-Bois, France) and were either mounted in OCT embedding compound (00411243, Labonord, Templemars, France) and frozen at −80 °C, or dehydrated in ethanol and embedded in paraffin.

### Flow cytometry and cell sorting

Mammary tissue fragments were thawed and enzymatically dissociated as previously described^12^ to obtain a single cell suspension. Dissociated cells were incubated with the relevant antibodies for 20 min at 4 °C, washed and re-suspended in MACS buffer (130-091-222, Miltenyi Biotec, Paris, France) with 2% bovine serum albumin (130-091-376; Miltenyi Biotec) for flow cytometry analysis or cell sorting.

Flow cytometry was performed using a MACSQuant Analyzer 10 cytometer (Miltenyi Biotec). The controls and gating strategy used in the present study have been previously detailed^12^. Note that isotype control antibodies were used as negative controls in the flow cytometry experiment. Data were analyzed using MACSQuantify analysis software (Miltenyi Biotec) and results expressed in percentage of cells out of 20,000 events.

ALDH1 activity was measured in 500.000 cells with the Aldefluor kit (01700, Stem cell technologies, Grenoble, France) according to the manufacturer’s recommendations. Cells were then centrifuged at 250 G, re-suspended in Aldefluor assay buffer and labeled with antibodies against CD49_f_ and CD24.

For cell sorting, cells were incubated with the relevant antibodies for 20 min at 4 °C in the dark. Single live cells were gated by DAPI exclusion and sorted on a BD FACS ARIA II flow cytometer (BIOSIT CytomeTRI technical Platform – Villejean Campus, Rennes, France). Sorted cells were centrifuged at 300 G for 5 min at 4 °C and stored at −80 °C. The antibodies used are described in the Supplementary Table S1.

### Mammosphere assays

Three-dimensional cell culture was performed on 48-well plates coated with Matrigel (356230, Life Sciences, Amsterdam, Netherlands). Wells received 100 µL of Matrigel and plates were left for 30 min at 37 °C for Matrigel gelation. Suspensions of sorted cells were centrifuged at 300 G during 5 min (4 °C) and the cell pellets were resuspended in Epicult medium (Epicult Basal medium, 05602, Stem cell technologies) supplemented with Epicult B supplement (05602, Stem cell technologies), L-Glutamine (2 mM final concentration), hydrocortisone (0.48 µg/mL final concentration) and 2% of Matrigel. Cells were plated at a concentration of 100.000 cells/well and cultured in Epicult medium for 7 days. Cells were observed at 10 × magnification using phase contrast microscopy (Zeiss Axio; Zeiss France, Fougères, France) and images were digitalized. Mammosphere mean diameters were determined from at least 12 frames per conditions using the Zen analysis software (Zeiss, France).

### Protein extraction and Western Blotting

Proteins were extracted from sorted cell populations, quantified using the BCA assay kit (23227, Thermo Fisher, Illkirch, France) and analyzed by Western blotting as previously described^42^, except that the amount of loaded protein was reduced to 2.5 µg. ECL signal was digitalized using the ImageQuant LAS4000 Imager digital system (GE Healthcare, Velizy-Villacoublay, France) and quantified with the ImageQuant TL software (GE Healthcare). Transferred proteins on PVDF membrane were stained using Coomassie brilliant blue R-250 (161-0436, Biorad, Marnes-la-Coquette, France) to normalize data. Coomassie blue stained PVDF membrane were digitized and total protein in each track was quantified as described above for the ECL signal. ECL signals were expressed as the percentage of total transferred proteins. The antibodies used are described in Supplementary Table S1.

### mRNA extraction and quantitative PCR

RNA extraction was performed using the Nucleospin RNA XS kit (740902, Macherey-Nagel, Hoerdt, France) according to the manufacter’s instructions. Reverse transcription and quantitative PCR were performed as previously described^12^. Raw cycle threshold (Ct) values obtained from StepOne Software version 2.3 (Applied Biosystems) were transformed into quantities using the delta Ct method. This calculation of Delta Ct (Ct of target gene/Ct of reference gene) was chosen to highlight the level of expression for each sub-population considered as independent sub-populations. The endogenous control gene, the Ribosomal Protein Large P0 (*RPLP0)*, was selected as the most stable gene within a panel of 3 genes (*18S rRNA*, *Ribosomal Protein S5* and *RPLP0*) using the Normfinder algorithm. The primers used in this study are described in Supplementary Table S2.

### Histological and immunohistochemical staining

Hematoxylin and eosin staining were performed on paraffin sections (8 µm) after rehydration as previously described^12^. CD49_f_ and CD24 immunostaining (see below) were performed on frozen sections (5 µm) mounted on Superfrost Plus slides (4951PLUS4, Thermo Fisher). CD10 immunostaining was done on paraffin sections (8 µm) as previously detailed^12^ with the following modifications. After deparaffinization, slides were first incubated with 50 mM ammonium chloride (A0171, Sigma-Aldrich) for 10 min and then with 0.1% Sudan black B (S2380, Sigma-Aldrich) in 70% ethanol for 20 min to quench the autofluorescence of immune cells. Slides were then rinsed with Tris-buffered saline (TBS) with 0.02% Tween-20 (P1379, Sigma-Aldrich). Tissue sections were then subjected to heat-induced epitope retrieval in 1 mM ethylenediaminetetraacetic acid (EDTA, E9884, Sigma-Aldrich), pH8, using a microwave at 800 watts for 2 × 5 min. For immunofluorescence analysis of sorted cells, cells were deposited on poly-lysine coated slides using cytospin at 800 rpm for 3 min and fixed for 30 min with 4% paraformaldehyde at room temperature. Sorted cells and sections from both frozen and paraffin-embedded tissue were then permeabilized with 0.25% Triton X-100 (T9284, Sigma-Aldrich). Nonspecific-antibody binding was blocked with 2% bovine serum albumin (A2153, Sigma-Aldrich) in TBS. Tissue slices were then sequentially incubated with primary and secondary antibodies (Table S1) at 37 °C for 1h30 and 45 min, respectively. After washing, nuclei were counterstained with Hoechst 33342 (14533, VWR) at 1 µg/mL for 2 min. Slides were mounted using Vectashield mounting medium (H-1000; Vector Laboratories, Burlingame, CA). Images were obtained with either an E400 Nikon microscope (Nikon France, Le Pallet, France) for the CD49_f_ and CD10 staining using the NIS-Elements BR4.20.00 software (Nikon) or an Apotome and Zen software (Zeiss france) for CD24 (Fig. 1), KRT7, KRT14, KRT8 and CD10 staining of sorted cells.

### Statistical analysis

Data were expressed as means ± SEM. PCR and cytometry results were subjected to an analysis of variance (ANOVA) using the R Studio software. Post-hoc Tukey pairwise comparisons were used. Different letters in Fig. 3 and Table 1 indicate significant differences (p < 0.05). For statistical analysis of Western blot results (Fig. S3) we used the non-parametric Mann-Whitney *U* test. Significant differences were considered at p < 0.05 and trends at p < 0.10.

## Electronic supplementary material

Supplementary information
