# Supplementary material for: Molecular signature of the putative stem/progenitor cells committed to the development of the bovine mammary gland at puberty
Source: Sci Rep. 2018 Nov 1;8:16194. doi: 10.1038/s41598-018-34691-2 (PMC6212573; doi:10.1038/s41598-018-34691-2)
Supplement: Supplementary file 1 — Supplementary information [file 41598_2018_34691_MOESM1_ESM.pdf]

Supplementary Information: Figures

Molecular signature of the stem/progenitor cells committed to the development of the bovine mammary gland at puberty

Laurence Finot, Eric Chanat and Frederic Dessauge

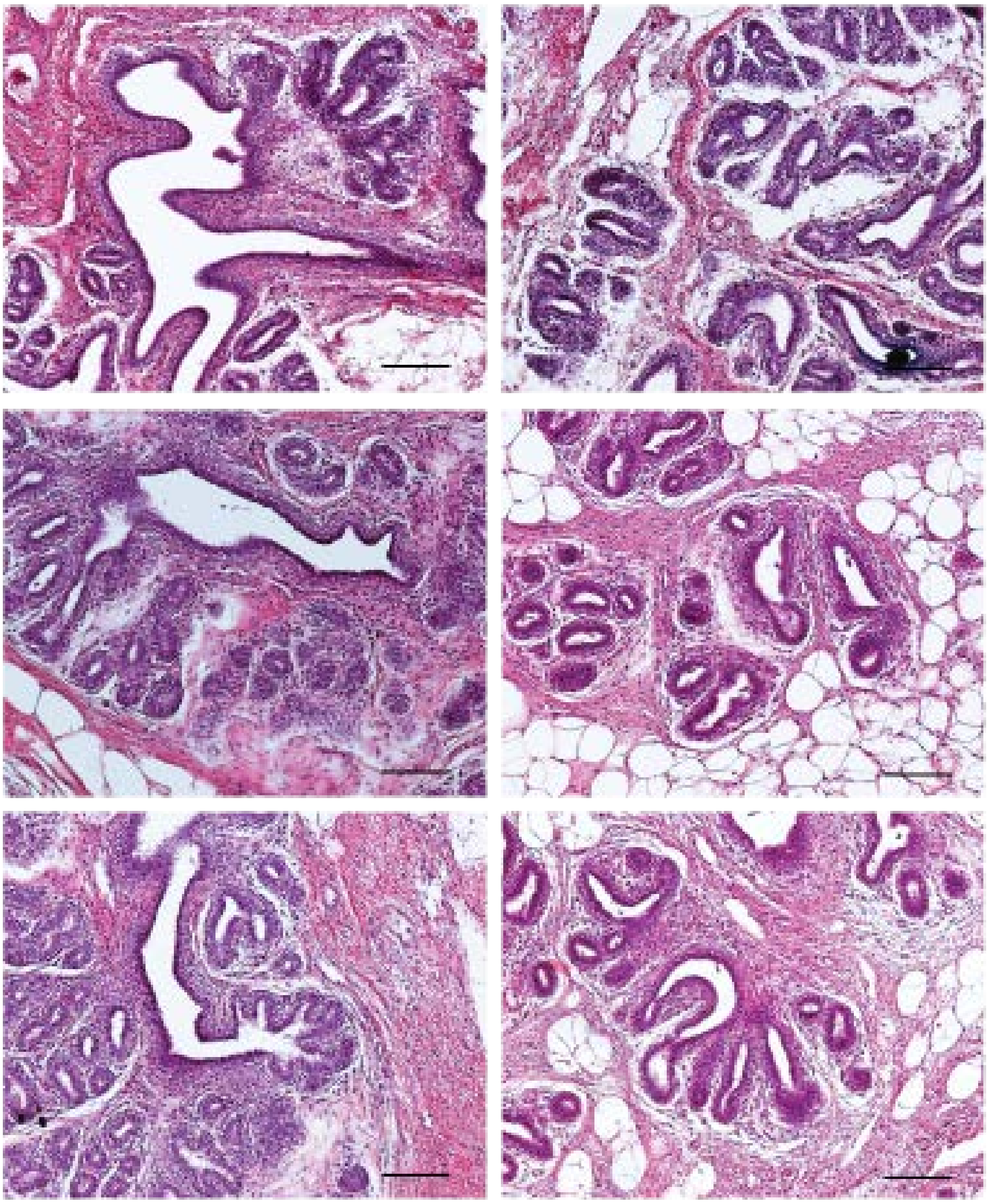

Figure S1. Structure of the bovine terminal ductal lobular unit at puberty. Representative sections from the mammary tissue of pubertal cows (two side-by-side images per animals) stained with hematoxylin and eosin are shown. These highlight complex developing parenchymal elements that are embedded in a dense stromal matrix and formed highly arborescent ductal and terminal ductal lobular units structures during parenchymal morphogenesis. Scale bars: 100 µm.

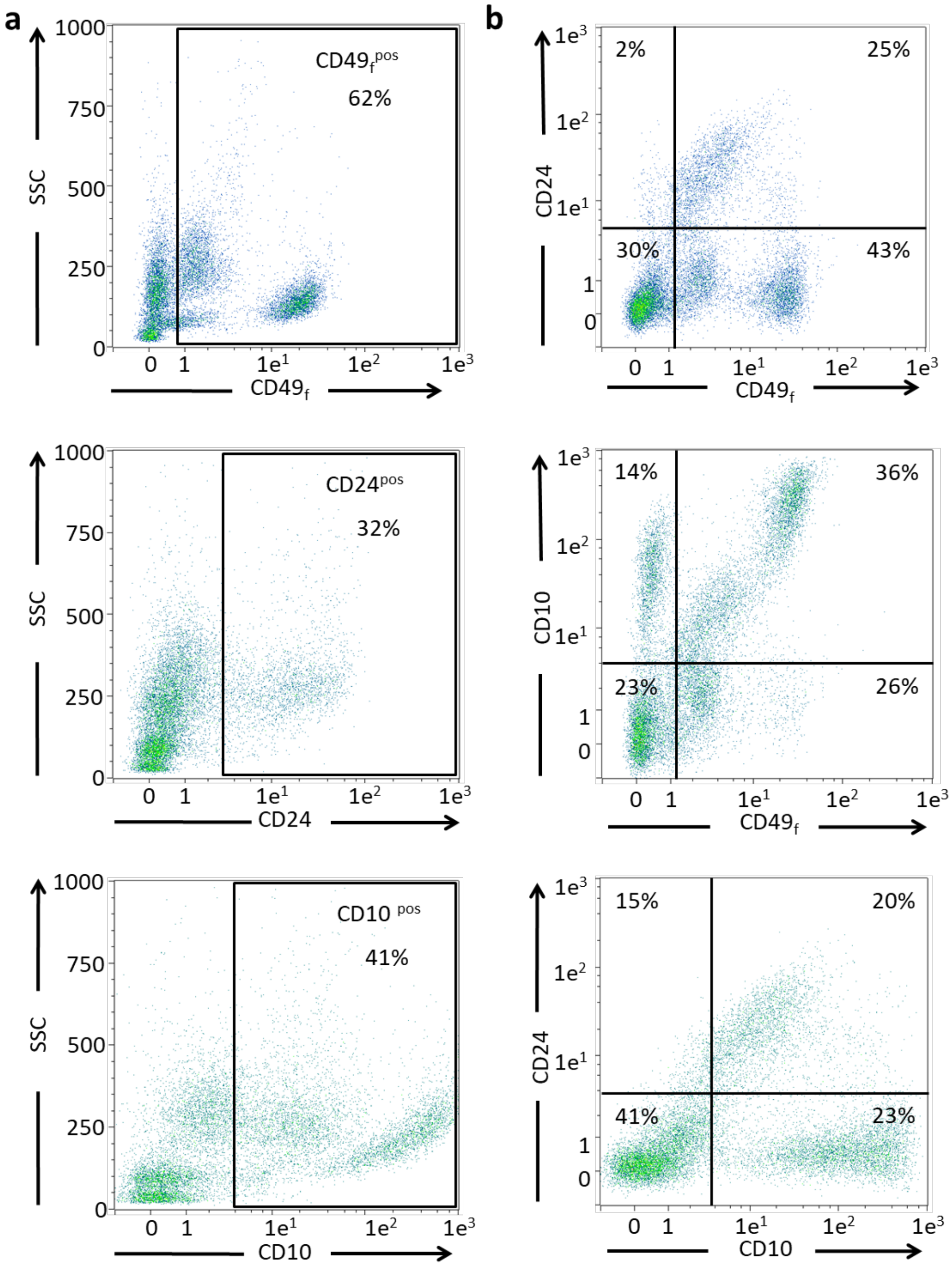

Figure S2. Distinct CD49$_f$, CD24 or CD10 expression characterizes the sub-populations of mammary epithelial cells that cohabitate within the developing bovine mammary epithelium. Dissociated cells from the mammary tissue of pubertal cows were either (**a**) stained with anti-CD49$_f$–FITC (CD49$_f$), anti-CD24-APC (CD24) or anti-CD10-PE Vio770 (CD10) antibodies or (**b**) co-stained with anti-CD49$_f$-FITC and anti-CD24-APC antibodies, anti-CD49$_f$-FITC and anti-CD10-PE Vio770 antibodies, or anti-CD10-PE Vio770 and anti-CD24-APC antibodies, and analyzed by flow cytometry. Each gating shows the positive cells. Sub-populations of epithelial cells were distinguished according to the intensity of the cell surface marker expression (low vs. high). The mean percentage of cells in each quadrant (percentage of total cells) determined from the flow cytometric profiles of three independent experiments (3 cows) is indicated. Abbreviation: SSC, Side Scatter light.

a

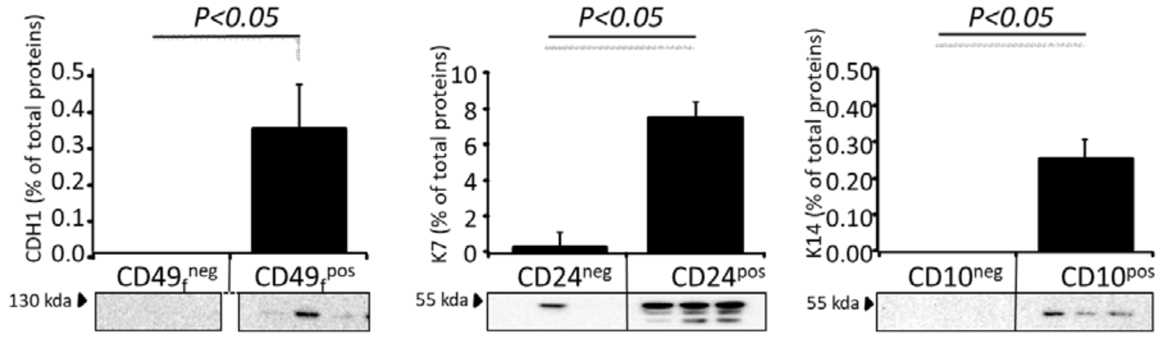

b

	CD49$_f^{neg}$	CD49$_f^{pos}$	p value	CD24 ^{neg}	CD24 ^{pos}	p value	CD10 ^{neg}	CD10 ^{pos}	p value
CDH1	0.00 ± 0.00	0.35 ± 0.21	p<0.05	0.04 ± 0.01	0.64 ± 0.34	p<0.05	0.17 ± 0.11	0.88 ± 0.30	p<0.10
CD10	1.11 ± 0.55	3.54 ± 0.63	p<0.05	1.63 ± 0.54	0.34 ± 0.02	p<0.05	0.00 ± 0.00	4.39 ± 1.06	p<0.05
aSMA	0.00 ± 0.00	1.50 ± 0.74	p<0.05	5.15 ± 1.17	0.32 ± 0.15	p<0.05	3.14 ± 0.56	15.20 ± 4.33	p<0.05
KRT14	0.00 ± 0.00	0.49 ± 0.12	p<0.05	0.15 ± 0.10	0.00 ± 0.00	ns	0.00 ± 0.00	0.27 ± 0.04	p<0.05
KRT19	1.04 ± 0.71	23.22 ± 7.99	p<0.05	7.13 ± 4.14	46.74 ± 3.46	p<0.05	13.22 ± 5.40	23.51 ± 2.06	ns
KRT18	0.00 ± 0.00	63.84 ± 25.41	p<0.05	2.76 ± 1.78	13.23 ± 1.41	p<0.05	3.36 ± 1.21	7.86 ± 1.32	ns
KRT7	0.10 ± 0.10	3.40 ± 1.09	p<0.05	0.74 ± 0.73	7.43 ± 0.59	p<0.05	0.55 ± 0.18	3.79 ± 0.97	p<0.05

Figure S3. The expression of CD49$_f$, CD24 and CD10 correlates to epithelial cell lineages of the bovine mammary gland. Cells dissociated from the mammary tissue of pubertal cows were stained and epithelial cell sub-populations were sorted according to the level of expression of either CD49$_f$, CD24 or CD10. Total protein extracts were analyzed by Western blotting with the indicated antibodies. The ECL signal was quantitated and the level of expression of the indicated proteins was expressed as mean percentage of total transferred proteins ±SEM, as described in the Method section. Three independent experiments were performed (3 cows). (**a**) Markers of the epithelial cell lineage distinguish the sorted cell sub-populations. The epithelial cadherin protein CDH1 was only present in the CD49$_f^{pos}$ cells, while the luminal marker protein KRT7 and the basal marker protein KRT14 were expressed in the CD24^{pos} and CD10^{pos} sub-populations, respectively. Cropped images from representative immunoblots are shown. Full-length immunoblot images are detailed in Figure S5. Relative molecular masses (kDa) are indicated. (**b**) Table summarizing western blotting data for protein markers of the epithelial cell lineages. Statistical analysis was performed using the Mann-Whitney U test. P value indicates significant differences ($p<0.05$), trends ($p<0.1$) and non-significant (ns) differences. Abbreviations: CDH1, E-cadherin; αSMA, alpha Smooth Muscle Actin; KRT14, Keratin 14; KRT19, Keratin 19; KRT18, Keratin 18; KRT7, Keratin 7.

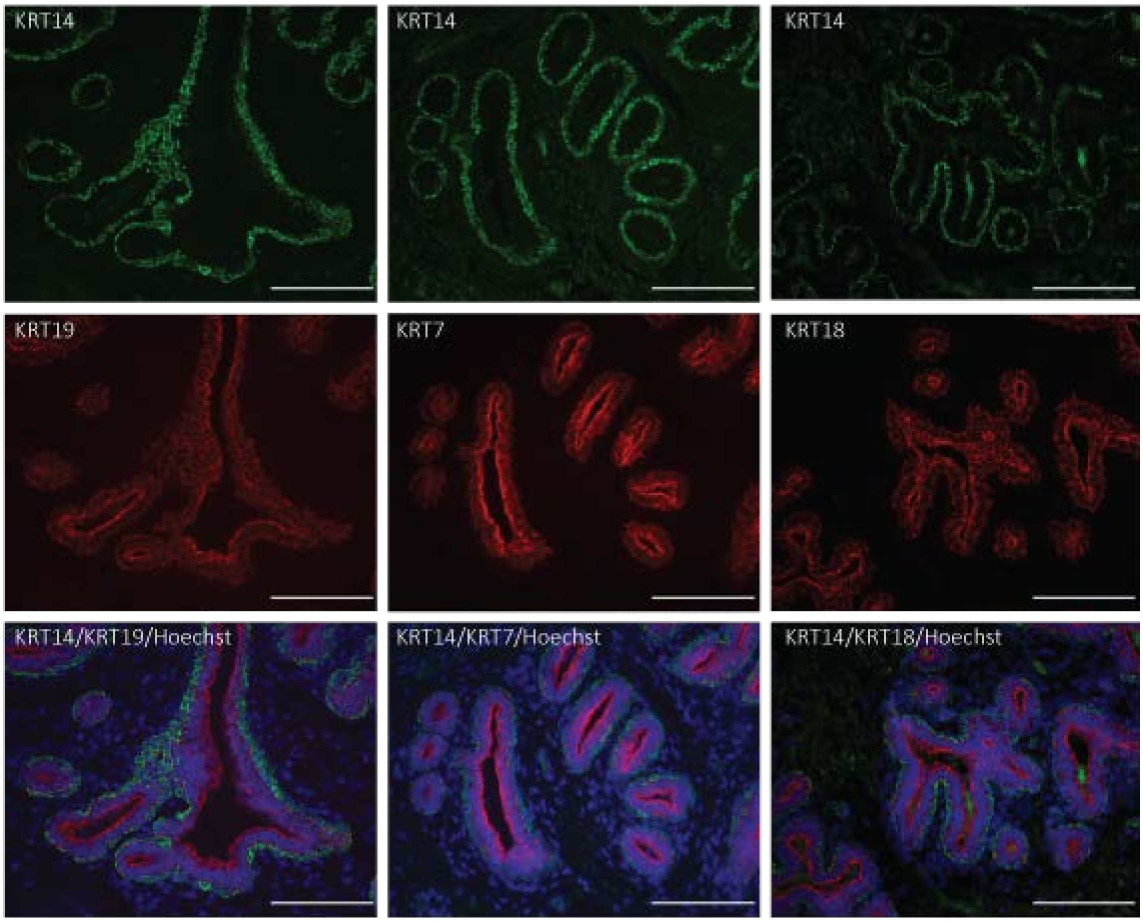

Figure S4. *In situ* localization of keratins demonstrates their lineage-specificity in the developing mammary tissue. Cryo-sections from the mammary tissue of pubertal cows were processed for immunofluorescence for the indicated antigens. Nuclei were counterstained with Hoechst 33342. Keratin 14 (KRT14) was predominantly expressed in basal cells (upper panels, green) whereas KRT19, KRT7 and KRT18 were expressed in luminal cells (middle panels, red). Relative localization of keratins was obtained by image merging of the indicated anti-keratin antibodies (lower panels, color-coded to match the fluorophore). Images are representative of 3 cows. Scale bars, 100µm.

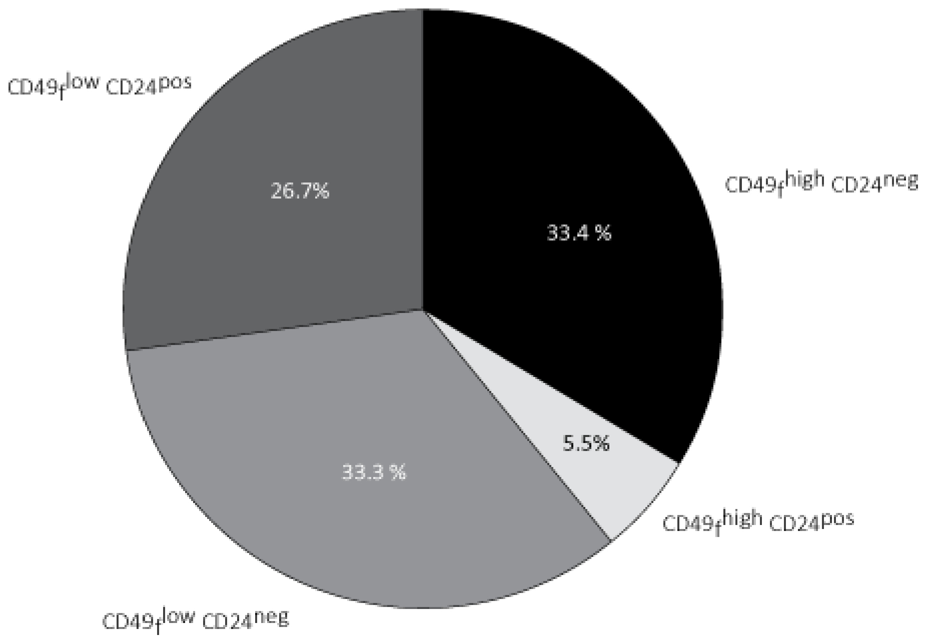

Figure S5. Proportion of each sub-population composing the epithelial cell fraction of the bovine mammary tissue at puberty. Cells dissociated from pubertal bovine mammary tissue were stained with anti-$CD49_f$ ($CD49_f$) and anti-CD24 (CD24) antibodies, and analyzed by flow cytometry. The number of cells in each sub-population of epithelial cells is expressed as the percentage of the total $CD49_f^{low}$ plus $CD49_r^{high}$ cells.

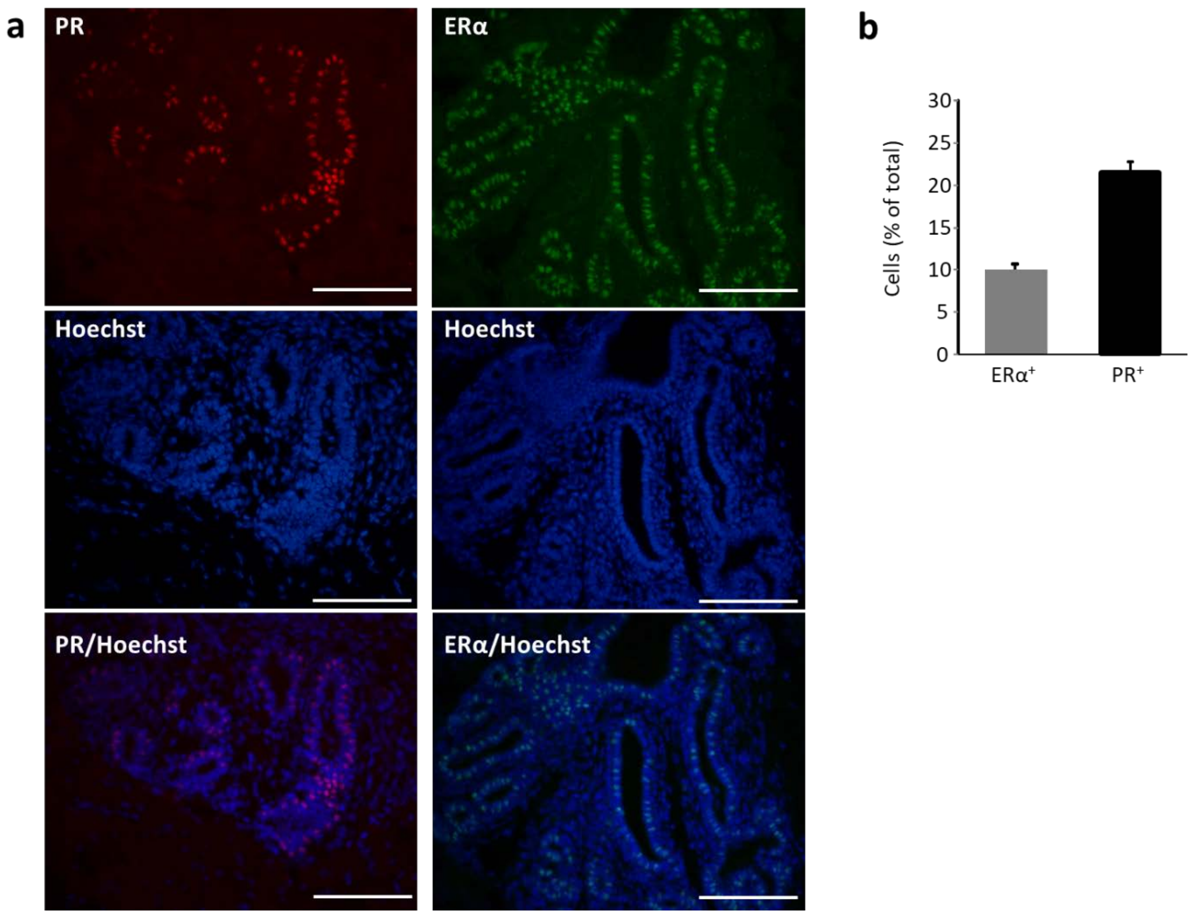

Figure S6. *In situ* localization of the cells expressing the receptors for progesterone and estradiol in the developing mammary tissue. Cryo-sections from the mammary tissue of pubertal cows were processed for immunofluorescence for the progesterone receptor (PR) and estradiol receptor alpha (ERα). Nuclei were counterstained with Hoechst 33342. **a**) A large number of the epithelial cells expressing the PR (left panel, red) and ERα (right panel, green) are located in the inner layer of the mammary structures. Images are representative of 3 cows. Scale bars, 100µm. **b**) Quantitation of the cells expressing PR and ERα within the mammary tissue. Results were obtained with six images per animal from 3 pubertal cows. Results are expressed in percentage ±SEM of stained cells (PR and/or ERα) relative to the total number of cells counterstained with Hoechst 33342.

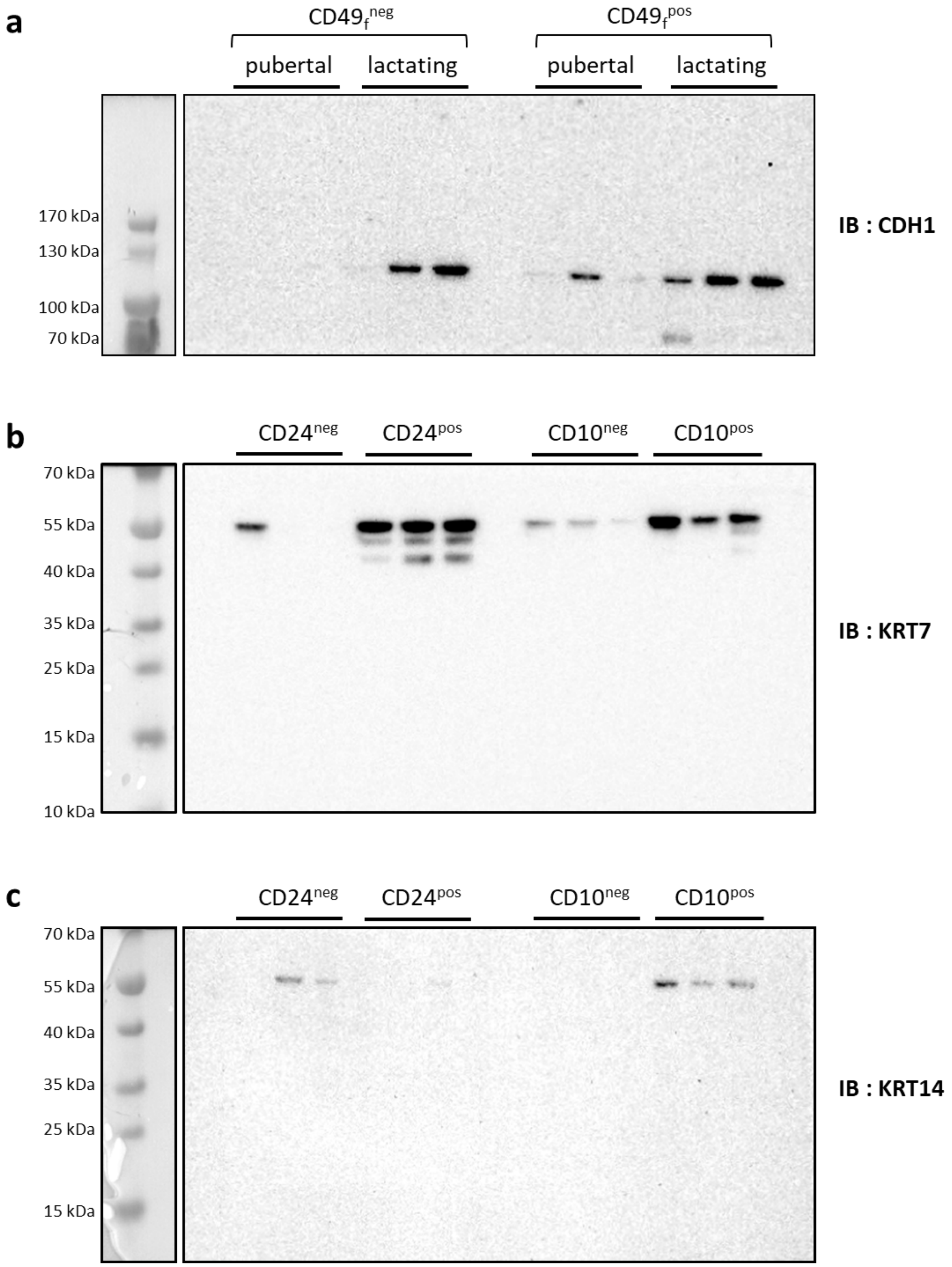

Figure S7. Full-length immunoblot images of Figure S3a.

Supplementary Information: Table

Molecular signature of the stem/progenitor cells committed to the development of the bovine mammary gland at puberty

Laurence Finot, Eric Chanat and Frederic Dessauge

Table S1. List of antibodies used for flow cytometry (FACS), Western Blotting and immunofluorescence analyses.

Antigen	Antibody	Manufacturer	Reference	Dilution (Application)
CD10	CD10-PE-Vio770, human (clone 97C5)	Miltenyi Biotec	130-100-421	1:10 (FACS)
Isotype control	Mouse IgG1-PE-Vio770	Miltenyi Biotec	130-096-654	1:10
	Mouse (clone 56C6)	Dako	M7308	1:200 (IF) / 1:2500 (WB)
CD24	CD24-APC, mouse (clone M1/69)	Stem Cell	60099AZ.1	1:10 (FACS)
Isotype control	Rat IgG2b-APC	Stem Cell	60077AZ.1	1:10
	CD24-FITC, mouse (clone M1/69)	Miltenyi Biotec	130-102-731	1:25 (IF)
$CD49_f$	$CD49_f$-FITC, human and mouse (clone GoH3)	Miltenyi Biotec	130-097-245	1:10 (FACS) / 1:25 (IF)
Isotype control	Rat IgG2a-FITC	Miltenyi Biotec	130-102-653	1:10
	$CD49_f$-PE, human and mouse (clone GoH3)	Miltenyi Biotec	130-100-096	1:10 (FACS)
Isotype control	Rat IgG2a-PE	Miltenyi Biotec	130-102-654	1:10
A-Smooth Muscle (αSMA)	Mouse (clone 1A4)	Santa Cruz	SC32251	1:2500 (WB)
E-cadherin (CDH1)	Mouse (clone CY-90)	Dako	M3612	1:2500 (WB)
Estrogen Receptor alpha (ERα)	Rabbit (clone HC-20)	Santa Cruz	SC543	1:100 (IF)
Keratin 7 (KRT7)	Mouse (clone 5F282)	Santa Cruz	SC70936	1:2500 (WB) / 1 :100 (IF)
Keratin 8 (KRT8)	Mouse (clone H-40)	Santa Cruz	SC134484	1:2500 (WB) / 1 :100 (IF)
Keratin 14 (KRT14)	Goat (clone C-14)	Santa Cruz	SC17104	1 :100 (IF)
Keratin 18 (KRT18)	Mouse (clone NCH38)	Sigma-Aldrich	C8541-.2ML	1:2500 (WB)
Keratin 19 (KRT19)	Mouse (clone b170)	Leica Biosystems	NCL-CK19	1:2500 (WB)
Progesterone Receptor (PR)	Mouse (clone PR10A9)	Beckman Coulter	PN IM1546	1:200 (IF)

Supplementary Information: Table

Molecular signature of the stem/progenitor cells committed to the development of the bovine mammary gland at puberty

Laurence Finot, Eric Chanat and Frederic Dessauge

Table S2. List of primers used in quantitative PCR

Gene	Accession N°	Forward primer (5'→3')	Reverse primer (5'→3')	Product size	DOI
ALDH1		CCTTGCATTGTGTTTGCTG	AACACTGGCCCTGGTGATA	85	10.1371/journal.pone.0030113
ELF5		ATACTGGACGAAGCGCCACGTC	ACTCCTCCTGTGTCATGCCGCA	134	10.1111/jpn.12039
ER α	NM_001001443.1	CAGGAGGAAGAGCTGTCAGG	ATCATCTCTCTGGCGCTTGT	125	
KRT 14	NM_001166575.1	TGATCAGCAGCGTGGAAGAG	TGATCAGCAGCGTGGAAGAG	164	
KRT 19	NM_001015600.3	GGCGGGCAACGAGAAGC	CGAGAATCTGGTCCCGCAG	200	
KRT 18	NM_001192095.1	GCGAGAAGGAGACCATGCAA	AGAATTTGCAAAAATCTGAGCCCT	197	
KRT 7	NM_001046411.1	GCACGCTCATCCTACGGG	AGAAACCGCACCTTGTCGAT	185	
NOTCH1		AACGAGTTCGTGTGCGAGT	GTTCTTGCAGGGTGTGCTT	90	10.1371/journal.pone.0030113
PROCR	NM_174437.1	CTTGAAAGGAAGCCAAACAGGC	TGGAGAGAATCAACACCGCC	136	
PR	XM_583951.3	TGCAGGACATGACAACAGCA	TTCCGAAAACCTGGCAGTG	123	
PRLR (long)	XM_010816795.2	CTGCTGGAGAAGGGCAAGTCCGAA	GTTCTTTGGAGGGGCGTGGCA		
18S rRNA	DQ066896.1	CAAATTACCCACTCCCGACCC	AATGGATCCTCGCGGAAGG	114	
R PLP0		CAACCCTGAAGTGCTTGACAT	AGGCAGATGGATCAGCCA	227	10.1016/j.vetimm.2006.09.012
RPS5	BC102374.1	GGAACATCAAGACCATTGCCG	GCGTAGGAATTGGAGGAGCC	76	
Vimentin	NM_173969	CAAGTCCAAGTTTGCTGACC	TCATGTTCTGAATCTCATCCTG	266	
